# Instrument for assessing surgical skills related to performing a vascular anastomosis

**DOI:** 10.1590/1677-5449.202400312

**Published:** 2025-02-14

**Authors:** Gabriela de Oliveira Buril, Joseph Monteiro de Carvalho, Vânia dos Santos Nunes-Nogueira

**Affiliations:** 1 Universidade Federal de Pernambuco – UFPE, Departamento de Cirurgia Vascular, Recife, PE, Brasil.; 2 Secretaria de Saúde do Distrito Federal, Brasília, DF, Brasil.; 3 Universidade Estadual Paulista – UNESP, Botucatu, SP, Brasil.

**Keywords:** educational measurement, vascular surgery, internship and residency

## Abstract

Technical skill is one of the most important components of surgical competence and must be thoroughly learned and developed over the course of surgical residency. However, there are still few instruments for directly appraising and quantifying surgical skill and the majority of centers rely on the subjective observations of tutors. The objective of this study was to conduct a scoping review on the subject of instruments for appraisal of surgical skills available for assessment of vascular anastomosis performed by vascular surgery residents. A review was conducted using the following search terms: “educational measurement”, “vascular surgery”, and “internship and residency”. The initial search results comprised 616 articles. After application of the eligibility criteria, just four studies were included in the review, only one of which was conducted in Brazil. Three of the studies used Objective Structured Assessment of Technical Skill (OSATS) to assess residents. However, all used separate and different supplementary checklists for evaluation of specific skills related to anastomoses. Appropriate appraisal and precise feedback during residency training are an extremely important part of residents’ training. Systematic and objective feedback enables errors to be corrected and skills to be polished. Since there is no instrument for assessment of the surgical skills needed to perform anastomosis, after completion of the review a rating instrument was proposed focused on this important skill that vascular surgery residents must acquire. The instrument comprises a compilation of the instruments reviewed and includes a proposed checklist for use in real-world settings.

## INTRODUCTION

Technical skill is one of the most important components of surgical competence and, as such, must be taught and assessed over the course of surgical residency. During a surgical procedure, 75% of the process is dependent on decision-making and 25% is dependent on dexterity.^1^ The two are directly linked and interdependent. Initially, decision making is to a great extent dependent on theoretical knowledge, which can be adequately assessed by oral or written tests, as has been clearly demonstrated. However, there are few objective methods for judging surgical skill (dexterity).^1^ To date, this skill is still being appraised subjectively by faculty, both here in Brazil and in most parts of the world.

Vascular anastomosis is a complex procedure that demands multiple, interrelated technical skills.^1^ A poorly placed stitch or a poorly executed knot can result in a poor suture, with potential blood loss or vascular thrombosis, which can even result in death.^2^ It is extremely necessary that vascular surgeons perform this procedure perfectly, because a well-executed suture and hemostasis are crucial steps in successful surgery.^3^ Moreover, a badly placed stitch can provoke thrombosis of a vessel, leading to loss of a limb or resulting in thrombosis of a hemodialysis access, for example. To execute an anastomosis, the surgeon must: plan the sequence of steps needed to complete the task; arrange the patient’s body and room lighting to enable good visualization of the surgical field; isolate the vessels to be anastomosed; move the patient’s body and arm to facilitate placement of stitches; perforate tissues at the correct angles for the needles; position all stitches with correct distance and tension to prevent blood loss, while causing the minimum possible damage to tissues.^1^

The proper time to learn how to perform vascular anastomosis correctly is during surgical residency. Initially, training should begin in simulation suites, using the most realistic simulators possible. Later, when the resident has already acquired the knowledge and skills needed, they will move on to real procedures. In the operating room, during a procedure on a patient, teaching and appreciation of knowledge and technical skills occur at brief moments, offering fleeting opportunities for learning and allowing little time for reflection. This is because there is not always a place for teaching calmly during surgery. The teaching physician’s attention is divided, in addition to the practice of teaching, between concern for the patient; thinking about the next steps; and worrying about anything that has evaded her or his control. All of these demands can interfere with the tutor’s ability to transfer knowledge and assess the resident.^3,4^ As a result, in order to avoid compromising the safety of a patient who is undergoing a given procedure, the teaching process is very often not conducted in the ideal manner.

When the objective is to assess a surgical skill, there is a need for objective, validated, and reproducible instruments that can be employed globally. If this were the case, it would be possible to appraise each resident individually and his or her development over the course of their training, in addition to enabling comparison of the results of different centers.^4^ Reproducibility is a necessary quality for any assessment instrument that is intended for widespread use.^3^ Reproducible instruments would enable assessment both of individual residents and also of the centers where they are being trained, standardizing surgery residency training in worldwide.^3^

For residents, formal and less subjective appraisal of technical skill offers several advantages. The first advantage is educational appraisal, providing the resident with objective feedback on the current status of their surgical skills. In possession of objective knowledge about their qualities and, most importantly, their deficiencies, residents will have the opportunity to address them. This feedback must be provided immediately, during the course of the medical residency, in time for the residents to be able to correct their failings and perfect their qualities. The second advantage is that an objective criterion makes it easier to judge when a resident is fit to move up to the next level of training, proceeding to the next level of proficiency or to the next year of the course, or is ready to be awarded the title of specialist.^5^

In 1997, Martin et al.^6^ constructed and validated an assessment instrument for general surgical skills, called the Objective Structured Assessment of Technical Skill (OSATS). This instrument has since been used for several surgical specialties, such as abdominal surgery, laparoscopy, and even vascular surgery, and has been adopted worldwide. The OSATS criteria are described in Table 1. In Brazil, OSATS was validated for use in appraisal of residents on surgical medical residency programs in 2020, by Campos et al.^7^ However, to date, we are unaware of any validated instruments specifically for assessment of vascular surgery surgical skills, whether in Brazil or globally.

**Table 1:** Details of the global scoring system for Objective Structured Assessment of Technical Skill (OSATS).

	1	2	3	4	5
Respect for tissue	Frequently used unnecessary force on tissue or caused damage by inappropriate use of instruments.		Careful handling of tissue but occasionally caused inadvertent damage.		Consistently handled tissues appropriately with minimal damage.
Time and motion	Many unnecessary moves.		Efficient time/motion but some unnecessary moves		Economy of movement and maximum efficiency.
Instrument handling	Repeatedly makes tentative or awkward moves with instruments.		Competent use of instruments although occasionally appeared stiff or awkward.		Fluid moves with instruments and no awkwardness.
Knowledge of instruments	Frequently asked for the wrong instrument or used an inappropriate instrument.		Knew the names of most instruments and used the appropriate instrument for the task.		Obviously familiar with the instruments required and their names.
Use of assistants	Consistently placed assistants poorly or failed to use assistants.		Good use of assistants most of the time.		Strategically used assistant to the best advantage at all times.
Flow of operation and forward planning	Frequently stopped operating or needed to discuss next move.		Demonstrated ability for forward planning with steady progression of operative procedure.		Obviously planned course of operation with effortless flow from one move to the next.
Knowledge of specific procedure	Deficient knowledge. Needed specific instruction at most operative steps.		Knew all important aspects of the operation.		Demonstrated familiarity with all aspects of the operation.

Source: Martin et al.^6^

The objective of this review was to determine how appraisal of surgical skills related to vascular anastomoses is described in the global literature. There is wide recognition of the importance of standardizing residency training, of analyzing centers that provide residencies, of providing residents with sufficient and precise feedback during their training and, finally, of judging whether they are ready to proceed to the next stage of training, to perform procedures with the minimum of help, to proceed with residency, or to be awarded the title of specialist.

Prior to initiating this review, a preliminary search was run on PubMed, which identified no current or ongoing scoping reviews on the subject.

## METHODS

A scoping review was conducted according to the methodology proposed by the Joanna Briggs Institute (JBI) for scoping reviews.^8^

The search strategy was tailored to the PubMed electronic database. The following index terms and their synonyms were used to identify eligible studies: “internship and residency”, “vascular surgical procedures”, and “educational measurement”. The term “Objective Structured Assessment of Technical Skill”, with the acronym “OSATS” was also included. The initial search identified 612 articles and another four articles identified by manual searches were added later.

Studies were selected for inclusion that met the eligibility criterion encompassed by the PCC acronym described below:

*Participants (P)* – Studies were included that recruited as participants eligible physicians enrolled on vascular surgery residencies. Physicians were defined as residents if they were enrolled on postgraduate training programs in vascular surgery, in the form of specialization courses, characterized by in-service training, under the responsibility of health institutions, universities or otherwise, and led by eminently qualified professional physicians, in terms of both ethical and professional qualifications.
*Concept (C) –* The concept studied is methods for appraisal of the technical skills of vascular surgery residents during construction of a vascular anastomosis.
*Context (C)* – The review considered studies investigating appraisal of technical skills in vascular surgery medical residencies, whether in the operating room or in simulation environments.

Studies were excluded if the participants were undergraduate students or physicians on health care courses that cannot be defined as medical residency. Studies were also excluded if they assessed general surgery residencies or residencies in surgical specialties other that vascular surgery. Finally, studies were excluded if they assessed skills other than construction of anastomosis, even if these fell within the area of vascular surgery.

This scoping review considered all available literature, including primary and secondary studies, letters, and guidelines, and any type of study design, including observational descriptive studies, cases series, individual case reports, and descriptive cross-sectional studies. Opinion texts and articles were also considered for inclusion in the scoping review. Studies published in English, Portuguese, or Spanish were included and no limit was applied to year of publication.

After the search strategy had been executed, all of the references identified were imported to the free web application RAYYAN. Titles and abstracts were then read by two independent reviewers to assess them against the predefined inclusion criteria for the review. Studies considered adequate were selected and the full texts were retrieved. The full texts of these articles were then analyzed in detail against the inclusion criteria, once more by the same two independent reviewers as in the previous step. Reasons for exclusion of studies that were found not to meet the inclusion criteria after reading the full text were recorded and reported in the review. Any disagreements between the two reviewers at each of the steps of the selection process were resolved by consensus or with the help of a third reviewer.

Data were extracted from articles and included in the scoping review by the two independent reviewers using a tool for data extraction developed for scoping reviews by JBI.^8^ The data extracted were: surname of lead author, year of publication, country, study design, participants, concept, context, outcomes analyzed, and main findings. Additional peculiarities of the articles were recorded under the heading “others”. This information was tabulated, in a manner aligned with the objective of the scoping review.

## RESULTS

**Figure 1:**
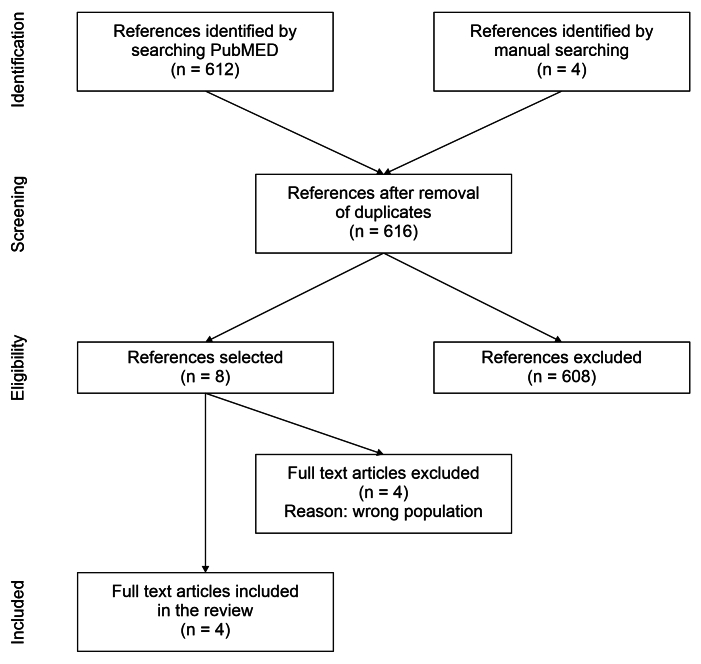
Flowchart illustrating selection of articles for the scoping review.

The search strategy returned 616 articles. The two reviewers selected eight of these studies for full text reading, four of which were included in the review.^1-3,5^ Thus, 612 studies that did not meet the eligibility criteria for the review were excluded. Table 2 lists the reasons for exclusion of the full text articles that were rejected.^9-12^Figure 1 summarizes the study selection process.

**Table 2:** Studies excluded after reading full text.

Authors	Year	Country	Reason for exclusion
Wilasrusmee et al.	2007	United States/TH	29 general surgery residents
Fann et al.	2008	United States	Heart surgery anastomosis simulator
Tavlasoglu et al.	2014	TR	Coronary anastomosis simulator for heart surgery residents
Shah et al.	2018	United States	21 general surgery residents

United States = United States of America; TH = Thailand; TR = Turkey.

Wilasrusmee et al.^9^ recruited 29 residents for their study, all of whom were general surgery residents. Fann et al.^12^ recruited eight heart surgery residents who performed 70 anastomoses on beating pig hearts, in a simulation environment. Tavlasoglu et al.^11^ simulated coronary anastomoses, recruiting 10 heart surgery residents and five fully trained heart surgery specialists. Shah et al.^10^ recruited 21 general surgery residents who were on vascular surgery placements.

The four studies included in the scoping review were published from 2007 to 2022. The studies all analyzed vascular surgery residents, but some of the studies also included other specialties.

One of the studies was conducted in the United States,^3^ two were conducted in the United States and Thailand in conjunction,^1^ and one was conducted in Brazil.^2^ All of the studies were conducted in simulation laboratories only.

With regard to the assessment instruments used in the studies, Duran et al.^3^ correlated two instruments for end-to-end arterial anastomoses, using a multiple anastomosis model, with 92 vascular surgery residents. One of these assessment instruments was the Objective Structured Assessment of Vascular Anastomosis (end-side), which comprises 25 assessment items and was classified as tiring and difficult to use in practice by evaluators. The evaluators preferred the other assessment instrument, which was the Global Rating Score (GRS), also based on the OSATS and employing a Likert scale for classification.^1,3^

Jogerst et al.^5^ employed the GRS for OSATS to assess vascular anastomoses constructed by residents.

Torres et al.^8^ used a low-fidelity simulator to train five vascular surgery residents to perform vascular anastomoses during the COVID-19 pandemic. The pandemic provoked a significant reduction in elective vascular surgery procedures. In response, these authors developed a simulator to train vascular surgery residents in surgical skills. Before assessment, the resident were trained in a workshop using the simulator. The residents were assessed using the OSATS, the final product was analyzed, and the operative time was analyzed. The original OSATS was used to appraise the trained residents (Table 1). For evaluation of the final product, the following elements were analyzed: size of the arteriotomy, anastomosis angle, spacing and depth of sutures, appropriate use of clamps, and leaks.^2^

For categorization of anastomosis leaks, Wilasrusmee et al.^1^ classified leak types as: 0 – no leaks; 1 – liquid dripping from the anastomosis; 2 – ejection of liquid from the anastomosis; and 3 – liquid bursting from the entire anastomosis. The authors also analyzed the degree of leakage and diameter of anastomosis, finding that a tenth of a millimeter increase in anastomosis diameter was associated with less leakage.

The outcomes assessed were: time taken for completion of anastomoses, leakage from sutures, caliber of anastomoses,^1^ analysis of the final product,^2^ OSATS grading,^2,3,5^ and checklist of performance during the procedure.^3,5^

Two studies assessed the outcome time taken for completion of anastomoses.^1,2^ Wilasrusmee et al.^1^ found that only time to completion of anastomosis, from start (incision into the structure) to completion of the anastomosis when the suture was cut after the final knot, exhibited a statistically significant difference between more experienced residents and beginners. The end-to-end type of anastomosis also influenced time for completion of the task, with borderline significance (p = 0.059).

Jorgerst et al.^5^ reported that GRS appraisal scores were correlated with residents’ experience. Internal consistency was 0.92. Reliability was confirmed by interexaminer correlation and internal consistency. However, correlations between tutors’ grades and residents’ self-assessment were low. In general, residents awarded themselves significantly higher grades than their tutors. The authors confirmed the applicability and reproducibility of the evaluation tool and made research suggestions for application of the tool in appraisals of performance in the operating room.

Jorgerst et al.^5^ also observed that ratings of technical skills performance were not consistent with one another and this inconsistency was partially attributable to lack of training and standardization of raters. Surgical skills were analyzed using videos recorded with GoPro® cameras fixed to the residents’ heads. The authors concluded that video reviews were more appropriate and consistent for rating residents than live observation during surgery. This is because there are other stressors present during in-person surgery that may change the resident’s appraisal. They employed Generalizability Theory (G Theory) to detect the extent to which rating variability in surgical education is attributable to raters and amenable to training.

Torres et al.^2^ observed improved OSATS (p = 0.049) scores and analysis of the final product (p = 0.049) after workshops and training with their low-fidelity simulation environment. Although time to completion of anastomosis improved, this factor was not statistically significant (p = 0.07) when results before and after anastomosis simulation training were compared.

Table 3 shows a summary of the studies included.

**Table 3:** Data extracted from the studies included in the scoping review.

Authors	Year	Country	Study design	Participants	Concept	Context	Outcomes assessed	Main findings	Others	Evidence level
Wilasrusmee et al.	2007	United States/ TH	Prospective, experimental, single-center	38 GS residents (1 VS)	Assessment of construction of anastomosis	Simulation environment	Suture time; leaks; caliber of anastomosis	Training time and type of anastomosis were only variables related to shorter completion time		1b
Duran et al.	2013	United States	Prospective, randomized, experimental, single-center	92 VS residents	Assessment of construction of anastomosis	Simulation environment	Performance checklist and OSATS for vascular anastomosis	Residents’ scores on the global scale correlated with their level of experience		1b
Jogerst et al.	2021	United States/ TH	Prospective, experimental, single-center	12 residents	Assessment of construction of anastomosis	Videos recorded in simulation environment	Global performance checklist for OSATS	Ratings exhibited inter-examiner variability. Need for training to achieve reproducibility	Residents’ specialties were not stated	1b
Torres et al.	2022	BRA	Prospective, controlled, experimental, single-center	10 VS residents	Assessment of construction of anastomosis	Simulation environment, after workshop	OSATS; time and analysis of final product	Residents’ results improved after the workshop	During the COVID-19 pandemic	1b

TH = Thailand; BRA = Brazil; GS = general surgery; VS = vascular surgery; OSATS = Objective Structured Assessment of Technical Skill.

## DISCUSSION

Appraisal of surgical skills is an extremely important part of surgeons’ training.^1-6^ In contrast with assessment of cognitive knowledge, for which multiple validated measurement instruments exist, the possibilities for rating surgical skills are lagging behind in terms of the possibilities for quantification.^1^ Vascular anastomoses are complex procedures and must be executed perfectly, because failures can compromise patient safety, resulting in bleeding or thromboses.^3^ This scoping review analyzed instruments used for assessment of vascular anastomoses constructed by (trained) vascular surgery residents globally. To date, there are no validated and unified instruments that enable global appraisal of centers and residents that would allow these data to be compared and standardized.^5^

All of the studies included in this review assessed residents in simulation environments only. There are no studies that have employed a real operating room as the setting for teaching and assessment, although that was the initial idea underpinning this review. Three of the four studies included in the review employed OSATS for part of the appraisal of surgical skills.^2,3,5^ OSATS is an instrument that has been widely studied and validated worldwide, including in Brazil.^7^ These studies also included additional performance checklists to understand the specific steps involved in vascular anastomosis, since OSATS does not cover some of these steps.^1^

The original OSATS assesses seven items and is described in Table 1. Each item is scored on a scale from 1 to 5, where 1 is worst performance and 5 is best.^6^ In contrast with published OSATS, which includes modifications for laparoscopy, modifications for rating an anastomosis have never been included in an OSATS specifically for this purpose.^13^

The studies employed parallel scales to make up for the deficiency in assessment of the specific steps of anastomosis. Torres et al.^2^ added analysis of the final product and time for completion of the procedure to the traditional OSATS. Jogerst et al.^5^ used an adapted and summarized OSATS. In turn, Wilasrusmee et al.^1^ did not use OSATS, choosing to assess three objectives: time to completion of the procedure, degree of leakage, and size of anastomosis. Wilasrusmee et al.^1^ found that the more training a resident had received, the more quickly he or she completed the entire task. This review judges this subject to be very important, but suggests that it should be analyzed separately from the checklist. This is because some anastomoses are more difficult to construct, requiring more time for completion, such as when it is necessary to fix plates or displace the intima, without this in itself constituting a deficiency in terms of time taken to complete.

Duran et al.^3^ were the only authors to employ a global assessment instrument, for appraisal of performance of end-to-end anastomoses. They added ratings for hemostasis, sutures and knots, and quality of the final product, and eliminated knowledge of instruments and knowledge of specific procedure. They themselves had suggested an even larger checklist, containing 25 items, but agreed in their own article that it would be tiring and reproducibility would be difficult to achieve.^3^

The authors of the present review agree that OSATS is a brilliant instrument for appraisal of surgical skills and is the best that has been published to date. However, the instrument must be adapted for the conditions of each surgical procedure. After performing this review, we suggest that the following items should be added to OSATS for appraisal of anastomosis in the operating room: size of anastomosis, degree of leakage, and time to completion of the procedure. Wilasrusmee et al.^1^ observed that each tenth of a millimeter increase in arteriotomy size was drastically related to reduction in leaks. As suggested by Duran et al.^3^ , we also consider it would be advantageous to include ratings of hemostasis (of all tissues, and not only the anastomosis itself), sutures and knots, and quality of the final product. However, in contrast to these authors, it is our impression that sutures and knots should be two separate items, since they assess different actions. This scoping review also suggests that the item specific knowledge about the procedure should be removed from the surgical skills assessment checklist, since this topic is better evaluated when administering cognitive tests of theoretical knowledge. Each skill should be rated using a five-point Likert scale.^13^Table 4 presents the instrument proposed in this scoping review. Trainees’ scores can range from 12 (worst performance) to 60 (best performance).

**Table 4:** Proposed instrument for assessment of surgical skills for vascular anastomoses.

	1	2	3	4	5
Respect for tissue	Frequently uses unnecessary force on tissue or causes damage by inappropriate use of instruments.		Careful handling of tissue but occasionally causes inadvertent damage.		Consistently handles tissues appropriately with minimal damage.
Surgical time and motion	Many unnecessary moves.		Efficient time/motion but some unnecessary moves		Economy of movement and maximum efficiency.
Hemostasis	Poor control of bleeding due to inappropriate method or causing tissue damage.		Some hemostasis lapses.		Rapid control of bleeding with appropriate method.
Instrument handling	Repeatedly makes tentative or awkward moves with instruments.		Competent use of instruments although occasionally appears stiff or awkward.		Fluid moves with instruments and no awkwardness.
Knowledge of instruments	Frequently asks for the wrong instrument or uses an inappropriate instrument.		Knows the names of most instruments and uses appropriate instrument for the task.		Obviously familiar with the instruments required and their names.
Use of assistants	Consistently places assistants poorly or fails to use assistants.		Good use of assistants most of the time.		Strategically uses assistant to the best advantage at all times.
Suture	Poor technique causing poor apposition of tissues and incorrect distance between stitches.		Suture reliable, but executed with incorrect movements.		Tissues well positioned, smooth technique, and correct distance.
Knots	Too few knots and loose knots		Sufficient number of knots, but knots loose		Six knots with sufficient tension to maintain the suture
Leakage	Anastomosis not completed	Liquid leaking from entire anastomosis	Liquid squirting from anastomosis	Liquid dripping from anastomosis	No leaks
Diameter of anastomosis	1x diameter of vessel		2x diameter of vessel		1.5x diameter of vessel
Flow of operation and forward planning	Frequently stops operating or needs to discuss next move.		Demonstrates ability for forward planning with steady progression of operative procedure.		Obviously planned course of operation with effortless flow from one move to the next.
Quality of final product	Well below expected standard and with observable failures.		Product has observable failures but could function adequately.		Excellent final product, with no imperfections and functioning well.
Total time for completion of anastomosis					

Minimum 12 points: worst rating. Maximum 60 points: resident has attained excellence in the task.

When starting to research this subject (assessment instruments for vascular surgery residents’ skills), the authors considered that there may be few publications on the subject. Once the research began, it was found that no syntheses of evidence, studies, or analyses had been published or was ongoing at the time of data collection. A scoping review was the literature review method chosen because its purpose is to seek and analyze knowledge gaps. Scoping reviews start from wider questions than systematic reviews and are intended to identify bottlenecks that merit future investigation. Moreover, they are also different from integrative reviews, which, according to Galvão and Pereira, combines studies with differing methodological natures to arrive at a more comprehensive understanding of a health care phenomenon. Widely used in the area of nursing, this type of review may include preclinical and clinical studies, and qualitative and quantitative studies among eligible publications. By means of compatibilization of information of varying types, the objective is to present a synthesis of different types of knowledge on a given subject.^14^ Integrative review methods have been standardized little and constitute a type of narrative review. In view of this, the present review was based on the JBI protocol, using the PRISMA Extension for Scoping Reviews (PRISMA-ScR) to report results, with tables presenting summaries of studies, accompanied by a descriptive synthesis.^14-16^

This study has several limitations. First, the scoping review was based on searches of just one database, PubMed, because the time available was extremely restricted. PubMed was chosen because it is the leading database used in medicine.

Second, all of the studies included were conducted in simulation environments. No studies were found that appraised construction of vascular anastomoses in real time, with patients, in the operating room. This is because there are additional stressors in real procedures that are not present in simulated environments: such as time of day; small differences in the instruments available; patients’ clinical problems; problems in other areas of the hospitals; unexpected problems during surgery; and *beeps* from cellphones, among others. Unfortunately, simulation laboratories are still rare in Brazil. There is a need to validate necessary instruments for appraisal of surgical skills in real-life operating room environments, which constitute the real situation at the majority of vascular surgery residencies in Brazil, which do not have simulation laboratories available. Assessment in real-life environments also offers the advantage of assessing an item that cannot be classified in simulated environments - hemostasis.^5^

Finally, even among the studies included, there was selection bias, since one of them only recruited one vascular surgery resident,^1^ and another, although it studied surgical residents, did not clearly identify which specialties were involved. However, since it used OSATS in its design and evaluation of vascular anastomoses by specialist vascular surgery surgeons, we included it among the studies reviewed.^5^

Assessments for surgical skills are very out of date when compared with assessments available for quantifying technical-cognitive knowledge. Few instruments for rating skills are available in the literature, and OSATS is the most widely used of those that exist.^6^ When a specific instrument for assessing construction of vascular anastomosis is needed, OSATS is often used with minor changes or additional checklists, but which have not been validated and are not employed globally.

It is necessary to develop an instrument to enable standardization of appraisal of vascular surgery residents and, consequently, their residency programs. In Brazil, there are no instruments developed for this purpose.

## CONCLUSIONS

The present study found that there are few studies in the global literature employing instruments for assessment of the surgical skills required for construction of a vascular anastomosis.

In Brazil, the present review found just one study employing OSATS, with the addition of time taken for completion of the anastomosis and analysis of the final product.

All of the studies reviewed used simulation environment as assessment setting and there are no studies that have used instruments for assessment of surgical skills in a real-life operating room setting.

This review proposes a new instrument for assessment of vascular anastomoses for use in the operating room.
