# Supplementary material for: Instrument for assessing surgical skills related to performing a vascular anastomosis
Source: J Vasc Bras. 2025 Feb 14;24:e20240031. doi: 10.1590/1677-5449.202400312 (PMC11841611; doi:10.1590/1677-5449.202400312)
Supplement: Translation

# Translation: Instrumento de avaliação de habilidades cirúrgicas relacionadas à realização de uma anastomose vascular

**DOI:** 10.1590/1677-5449.202400311

**Authors:** Gabriela de Oliveira Buril, Joseph Monteiro de Carvalho, Vânia dos Santos Nunes-Nogueira

**Affiliations:** 1 Universidade Federal de Pernambuco – UFPE, Departamento de Cirurgia Vascular, Recife, PE, Brasil.; 2 Secretaria de Saúde do Distrito Federal, Brasília, DF, Brasil.; 3 Universidade Estadual Paulista – UNESP, Botucatu, SP, Brasil.

**Keywords:** avaliação de desempenho profissional, cirurgia vascular, internato e residência

## Abstract

A habilidade técnica é um dos mais importantes componentes da competência cirúrgica, devendo ser bem aprendida e desenvolvida durante as residências cirúrgicas. Entretanto, a quantificação da habilidade cirúrgica ainda possui poucos instrumentos de julgamento diretos, valendo-se, na maioria dos centros, da observação subjetiva dos preceptores. O objetivo deste estudo foi realizar uma revisão de escopo sobre os instrumentos de avaliação das habilidades cirúrgicas existentes para avaliar a realização de uma anastomose vascular por residentes de cirurgia vascular. Uma revisão foi realizada utilizando os seguintes descritores: “*educational measurement*”, “*vascular surgery*” e “*internship and residency*”. Inicialmente, foram encontrados 616 artigos. Após aplicação dos critérios de elegibilidade, foram incluídos apenas quatro estudos nesta revisão, sendo apenas um desses brasileiro. Três estudos utilizaram o Objective Structured Assessment of Technical Skill (OSATS) para a avaliação dos residentes, entretanto, todos utilizaram uma lista de verificação separada e diversa associada para realizar o julgamento das habilidades específicas relacionadas à confecção de anastomoses. O adequado julgamento e a precisa devolutiva durante a formação do residente são de extrema importância para a formação desse. Quando se promove uma devolutiva sistematizada e objetiva, permite-se que erros sejam corrigidos e habilidades sejam lapidadas. Como não há um instrumento de avaliação de habilidades cirúrgicas para a confecção de anastomose, após esta revisão, foi sugerido um instrumento de gradação focado nessa importante habilidade que precisa ser adquirida pelo residente de cirurgia vascular. Para tanto, foi utilizado um compilado dos instrumentos utilizados e sugerida uma lista de verificação para ser utilizada num ambiente real.

## INTRODUÇÃO

A habilidade técnica é um dos mais importantes componentes da competência cirúrgica e, como tal, precisa ser ensinada e avaliada durante as residências de cirurgia. No ínterim da realização de um procedimento cirúrgico, 75% do processo depende da tomada de decisões e 25% da destreza^1^ . Ambos são diretamente ligados e dependentes um do outro. Inicialmente, a tomada de decisão depende, em grande parte, dos conhecimentos teóricos, e pode ser adequadamente avaliada por exames orais ou escritos já bem demonstrados. Já a avaliação de habilidade cirúrgica (destreza) ainda possui poucos métodos de julgamento objetivo^1^ . Ainda atualmente, essa habilidade vem sendo apreciada subjetivamente pelos preceptores, tanto no Brasil quanto em grande parte do mundo.

A anastomose vascular é um procedimento complexo que exige múltiplas habilidades técnicas agrupadas^1^ . Um ponto mal colocado ou um nó mal realizado podem resultar em uma má sutura, com potencial perda sanguínea ou trombose vascular, podendo levar até mesmo ao óbito^2^ . É extremamente necessário que os cirurgiões vasculares realizem esse procedimento com perfeição, pois uma sutura bem realizada e a hemostasia são passos cruciais para o sucesso de uma cirurgia^3^ . Além disso, um ponto mal posicionado pode ocasionar a trombose do vaso, levando à perda de membros ou à trombose de acesso para hemodiálise, por exemplo. Para realizar uma anastomose, o cirurgião precisa: planejar a sequência de passos para completar a tarefa; ajustar o corpo do paciente e a iluminação da sala para que possa observar bem o campo cirúrgico; isolar os vasos que serão anastomosados; mover o corpo e o braço do paciente para facilitar o posicionamento dos pontos; perfurar os tecidos com a angulação adequada das agulhas; posicionar todos os pontos com distância e força adequadas para evitar vazamento de sangue e causar o mínimo dano possível aos tecidos^1^ .

O momento propício para aprender a realizar uma anastomose vascular adequada é durante a residência cirúrgica. Inicialmente, esse aprendizado deve ocorrer em salas de simulação, utilizando simuladores o mais realistas possíveis. Posteriormente, quando o residente já tiver acumulado os conhecimentos e habilidades necessários, passa-se aos procedimentos reais. Na sala de cirurgia, durante um procedimento com o paciente, o ensino e a apreciação de conhecimentos e habilidades técnicas ocorrem em momentos breves, oferecendo oportunidades fugazes para aprendizagem e permitindo pouca reflexão. Isso porque, durante a cirurgia, nem sempre há espaço para um ensino tranquilo. A atenção do médico preceptor permanece dividida, além do ensino, entre: a preocupação com o paciente; o pensamento com os próximos passos e a preocupação com algo que fugiu ao controle. Todas essas demandas podem dificultar a entrega de conhecimento e avaliação do residente pelo preceptor^3,4^ . Então, para se evitar o comprometimento da segurança do paciente que está sendo submetido a determinado procedimento, muitas vezes o processo de ensino-aprendizagem não é conduzido da melhor forma.

Quando se busca avaliar uma habilidade cirúrgica, há a necessidade de existirem instrumentos objetivos, validados e reprodutíveis, que possam ser utilizados globalmente. Assim, seria possível avaliar o residente individualmente e acompanhar sua evolução durante o período de aprendizagem, além de comparar resultados de serviços entre si^4^ . A reprodutibilidade é uma qualidade necessária para qualquer instrumento de avaliação que pretenda ser utilizado difusamente^3^ . Com isso, seria possível realizar tanto a avaliação de um residente em si quanto de um serviço como um todo, uniformizando as formações dos residentes de cirurgia globalmente^3^ .

Para os residentes, um julgamento de habilidade técnica formal e menos subjetiva possui várias vantagens. A primeira vantagem é ser uma avaliação formativa, fornecendo ao residente uma devolutiva (*feedback)* objetiva sobre o estado atual das suas habilidades cirúrgicas. Sabendo objetivamente de suas qualidades e, principalmente, deficiências, o residente terá a oportunidade de resolvê-las. Essa devolutiva deve ser entregue imediatamente, ainda durante a residência médica, com tempo suficiente para que o residente possa corrigir suas falhas e aprimorar suas qualidades. A segunda vantagem é que, com um critério objetivo, torna-se mais fácil julgar e saber quando o residente está apto para ascender na formação, passando para o próximo grau de proficiência, para o próximo ano de curso ou recebendo o título de especialista^5^ .

Em 1997, foi criado e validado por Martin et al.^6^ um instrumento de avaliação de habilidades cirúrgicas em geral, chamado de *Objective Structured Assessment of Technical Skill* (OSATS). Esse instrumento foi utilizado posteriormente para várias especialidades cirúrgicas, como cirurgia abdominal, laparoscopia e até mesmo cirurgia vascular, sendo adotada no mundo inteiro. O OSATS encontra-se descrito na Tabela 1 abaixo. No Brasil, o OSATS foi validado para o uso em apreciações de residentes em programas de residência médica cirúrgica em 2020, por Campos et al.^7^ . Entretanto, até o momento, não se tem conhecimento de nenhum instrumento validado que avalie habilidades cirúrgicas específicas para cirurgia vascular, seja no Brasil ou no mundo todo.

**Tabela 1:** Escala detalhada de pontuação global para *Objective Structured Assessment of Technical Skill* (OSATS).

	1	2	3	4	5
Respeito pelos tecidos	Frequentemente usa força desnecessária nos tecidos, causando danos pelo uso inapropriado de instrumentos		Manejo cuidadoso dos tecidos, mas ocasionalmente causa danos inadvertidos aos tecidos		Consistentemente manuseia adequadamente os tecidos, com mínimo dano
Movimentação e tempo cirúrgico	Muitos movimentos desnecessários		Movimentos e tempo efetivos, mas com movimentos desnecessários		Economia de movimento, com o máximo de eficiência
Manuseio dos instrumentos	Faz tentativas repetidas ou movimentos inábeis com instrumentos		Uso competente de instrumentos, apesar de ocasionalmente aparentar grosseiro ou inábil		Movimentos fluidos com instrumentos hábeis
Conhecimento do instrumental	Frequentemente pede instrumento errado ou usa instrumento inapropriado		Sabe do nome da maioria dos instrumentos e usa o instrumento apropriado para a tarefa		Obviamente familiarizado com os instrumentos necessários e seus nomes
Utilização de assistentes	Posiciona mal os assistentes ou falha em seu uso		Bom uso dos assistentes na maioria do tempo		Utiliza o assistente estrategicamente bem em todo o tempo
Fluxo da cirurgia e planejamento de passos	Frequentemente interrompe a cirurgia ou precisa discutir o próximo passo		Demonstra habilidade de planejar o próximo passo com progressão estável da cirurgia		Obviamente planeja o curso da cirurgia sem esforço no passo a passo
Conhecimento específico do procedimento	Conhecimento deficiente. Precisa de orientação específica na maioria dos passos da cirurgia		Sabe todos os passos importantes da cirurgia		Demonstra familiaridade com todos os aspectos da cirurgia

Fonte: Martin et al.^6^ .

O objetivo desta revisão foi identificar como são conduzidas as avaliações de habilidades cirúrgicas relacionadas à realização de anastomoses vasculares existentes na literatura mundial. É reconhecida a importância de padronizar o treinamento dos residentes, analisar os serviços de residência, entregar a adequada e precisa devolutiva formativa para os residentes e, por fim, julgar se estão prontos para ascender no aprendizado, seja para realizar um procedimento com o mínimo de auxílio, progredir na residência ou receber o título de especialista.

Antes de iniciar esta revisão, foi realizada uma pesquisa preliminar na PubMed e nenhuma revisão de escopo atual ou em andamento sobre o tema foi identificada.

## MÉTODOS

Foi realizada uma revisão de escopo de acordo com a metodologia do Instituto Joanna Briggs (JBI) para revisões de escopo^8^ .

A estratégia de busca da pesquisa foi criada e adaptada à base de dados eletrônicos de saúde PubMed. Para procurar os estudos elegíveis, foram utilizados os seguintes termos índice e seus sinônimos: “*internship and residency*”, “v*ascular surgical procedures*” e “e*ducational measurement*”. Foi acrescentado o termo “*Objective Structured Assessment of Technical Skill*”, sob o acrônimo “OSATS”. Inicialmente, foram encontrados 612 artigos, posteriormente acrescidos de quatro artigos identificados através de busca manual.

Foram incluídos estudos que continham como critério de elegibilidade o acrônimo PCC descrito abaixo:

*Participantes (P)* - Foram considerados estudos que tiveram como participantes elegíveis médicos residentes em cirurgia vascular. Considerou-se como residentes aqueles médicos inscritos em programas de ensino de pós-graduação em cirurgia vascular, sob a forma de cursos de especialização, caracterizados por treinamento em serviço, funcionando sob a responsabilidade de instituições de saúde, universitárias ou não, sob a orientação de profissionais médicos de elevada qualificação ética e profissional.
*Conceito (C) -* O conceito estudado foi a forma de avaliar as habilidades técnicas dos residentes de cirurgia vascular durante a confecção de uma anastomose vascular.
*Contexto (C)* - Esta revisão considerou pesquisas sobre as avaliações de habilidades técnicas na residência médica de cirurgia vascular, seja em sala de cirurgia, seja em ambiente simulado.

Foram excluídos estudos cujos participantes eram alunos de graduação ou médicos em cursos da área da saúde que não se caracterizavam como residência médica. Também, foram excluídos estudos que avaliavam residentes de cirurgia geral ou de outras especialidades cirúrgicas que não a vascular. Por fim, foram excluídos os estudos que avaliavam outras habilidades que não a realização de uma anastomose, mesmo que estivessem dentro do tema da cirurgia vascular.

Esta revisão de escopo considerou qualquer literatura disponível, como estudos primários e secundários, cartas, diretrizes, bem como qualquer desenho de estudo, incluindo os estudos observacionais descritivos, séries de casos, relatórios de casos individuais e estudos transversais descritivos. Textos e artigos de opinião também foram considerados para inclusão nesta revisão de escopo. Foram incluídos estudos publicados em inglês, português e espanhol. Não houve restrição de ano de publicação.

Após utilizada a estratégia de busca, todas as referências identificadas foram importadas ao aplicativo gratuito da *web* RAYYAN. Os títulos e resumos foram então lidos por dois revisores independentes para avaliação dos critérios de inclusão previamente definidos a serem utilizados na revisão. Os estudos considerados adequados foram incluídos e recuperados na íntegra. Os textos completos dos artigos selecionados foram, então, avaliados detalhadamente em relação aos critérios de inclusão, novamente pelos dois revisores independentes da etapa anterior. Os motivos para a exclusão de estudos que, após a leitura do texto completo, não atenderam aos critérios de inclusão foram registrados e relatados na revisão. As divergências entre os revisores em cada etapa do processo de seleção do estudo foram resolvidas por consenso ou com a ajuda de um terceiro revisor.

Os dados foram extraídos dos artigos e incluídos na revisão de escopo por dois revisores independentes usando uma ferramenta de extração de dados desenvolvida pelo JBI para revisões de escopo^8^ . Os dados incluídos foram: sobrenome do primeiro autor, ano de publicação, país, desenho do estudo, participantes, conceito, contexto, desfechos avaliados e principais resultados. Em “outros”, foram acrescentadas curiosidades adicionais dos artigos. Essas informações foram apresentadas de forma tabular, de maneira alinhada com o objetivo desta revisão de escopo.

## RESULTADOS

A estratégia de busca resultou em 616 artigos. Desses, foram selecionados pelos dois revisores oito estudos para leitura na íntegra, dos quais quatro foram incluídos^1-3,5^ . Foram excluídos 612 estudos que não estavam de acordo com os critérios de elegibilidade da revisão. Os motivos da exclusão dos artigos lidos na íntegra estão na Tabela 2^9-12^ . O processo de seleção dos estudos está resumido na Figura 1.

**Tabela 2:** Trabalhos excluídos após leitura integral.

Autor	Ano	País	Motivo da Exclusão
Wilasrusmee et al.	2007	EUA/TH	29 residentes de cirurgia geral
Fann et al.	2008	EUA	Simulador de anastomoses em cirurgia cardíaca
Tavlasoglu et al.	2014	TR	Simulador de anastomoses coronarianas para residentes em cirurgia cardíaca
Shah et al.	2018	EUA	21 residentes de cirurgia geral

EUA = Estados Unidos da América; TH = Tailândia; TR = Turquia.

**Figura 1:** Fluxo de seleção dos artigos utilizados na revisão de escopo.

Wilasrusmee et al.^9^ incluíram no seu estudo 29 residentes, sendo todos de cirurgia geral. Fann et al.^12^ incluíram oito residentes de cirurgia cardíaca que realizaram 70 anastomoses em corações porcinos batendo, em ambiente de simulação. Tavlasoglu et al. realizaram simulação em anastomoses coronarianas e incluíram 10 residentes de cirurgia cardíaca e cinco especialistas em cirurgia cardíaca com formação concluída^11^ . Shah et al.^10^ incluíram no seu artigo 21 residentes de cirurgia geral que se encontravam no rodízio de cirurgia vascular.

Os quatro estudos incluídos nesta revisão de escopo foram realizados entre os anos de 2007 e 2022. As especialidades de residentes abrangidas nos estudos foram de cirurgia vascular, mas alguns dos estudos também incluíam outras especialidades.

Um dos estudos incluídos pertence aos EUA^3^ , dois foram realizados em conjunto entre os EUA e a Tailândia^1^ , e um foi realizado no Brasil^2^ . O ambiente compreendido nos estudos foi apenas o laboratório de simulação.

Em relação aos instrumentos de avaliação utilizados nos estudos, Duran et al.^3^ correlacionaram dois instrumentos para a realização de anastomoses arteriais término-terminal, utilizando um modelo de múltiplas anastomoses, com 92 residentes de cirurgia vascular. Um dos instrumentos de avaliação foi o *Objective Structured Assessment of Vascular Anastomosis (end-side), que* possuía 25 itens a serem avaliados, tendo sido classificado como cansativo e difícil de pôr em prática pelos avaliadores. Já o segundo instrumento de avaliação, que foi mais aceito pelos avaliadores, foi o Global Rating Score (GRS), também baseado no OSATS e utilizando a escala de Likert para classificação^1,3^ .

Jogerst et al.^5^ utilizaram GRSs para OSATS para avaliar as anastomoses vasculares realizadas pelos residentes.

Torres et al.^2^ utilizaram um simulador de baixa fidedignidade para treinar cinco residentes de cirurgia vascular em anastomoses vasculares durante a pandemia de covid-19. Em vigência da pandemia, houve uma redução significativa na realização de cirurgias vasculares eletivas. Em decorrência desse fato, os autores desenvolveram um simulador para treinar as habilidades cirúrgicas dos residentes de cirurgia vascular. Antes da avaliação, os residentes eram treinados em um *workshop* com o simulador. Os residentes foram avaliados utilizando o OSATS, uma análise do produto final e o tempo de procedimento. Foi utilizado o OSATS original para julgamento dos treinandos (Tabela 1)^8^ . Para a análise do produto final, foram analisados: tamanho da arteriotomia, ângulo da anastomose, espaço e profundidade das suturas, uso apropriado de clampes e vazamento^2^ .

Quanto ao vazamento das anastomoses, Wilasrusmee et al.^1^ classificaram os tipos de vazamento em: 0 – sem vazamento; 1 – líquido pingando pela anastomose; 2 – ejeção de líquido pela anastomose; 3 – líquido vazando por toda a anastomose. Os autores também realizaram uma análise do grau de vazamento e diâmetro da anastomose, e descobriram que um aumento de um décimo de milímetro na anastomose levou a menos vazamentos.

Em relação aos desfechos, foram avaliados: tempo de realização das anastomoses, vazamento das suturas, calibre das anastomoses^1^ , análise do produto final^2^ , preenchimento de OSATS^2,3,5^ e *checklist* do desempenho durante o procedimento^3,5^ .

No que se refere ao tempo para a realização da anastomose, dois trabalhos avaliaram esse desfecho^1,2^ . Wilasrusmee et al.^1^ acharam que o tempo de confecção da anastomose, do início (incisão na estrutura) ao término da sutura da anastomose e corte do nó final, foi o único com diferença estatística significativa entre os residentes mais experientes e os iniciantes. O tipo de anastomose, término-terminal, também influenciou o tempo de conclusão da tarefa, com significância limítrofe (p = 0,059).

Jorgerst et al.^5^ chegaram ao resultado de que as pontuações nas avaliações das GRSs correlacionavam-se com a experiência dos residentes. A consistência interna foi de 0,92. A confiabilidade foi confirmada por meio da correlação interavaliadores e consistência interna. Já a correlação entre as pontuações dos avaliadores preceptores e a autoavaliação dos residentes foi baixa. Em geral, os residentes atribuíram a si uma nota significativamente mais alta do que os avaliadores. Os autores confirmaram a aplicabilidade e reprodutibilidade da ferramenta de avaliação e sugerem pesquisas para aplicação dela no julgamento de desempenho na sala de cirurgia.

Jorgerst et al.^5^ notaram ainda que as classificações de desempenho das habilidades técnicas não foram consistentes umas com as outras e que parte dessa inconsistência foi decorrente da falta de treinamento e padronização dos avaliadores. Para as análises das habilidades cirúrgicas, foram utilizados vídeos realizados por máquinas GoPro® fixadas às cabeças dos residentes. Os autores chegaram à conclusão de que as revisões de vídeo são mais adequadas e consistentes para avaliar o residente do que a observação ao vivo durante a cirurgia. Isso porque, no momento da cirurgia ao vivo, existem outros estressores que podem modificar o julgamento do residente. Eles utilizaram ainda a Teoria da Generalização (Teoria G) para detectar quanto da variabilidade das pontuações nas avaliações de educação cirúrgica é atribuível aos avaliadores e passível de treinamento.

Torres et al.^2^ notaram uma melhora da pontuação do OSATS (p = 0,049) e da análise do produto final (p = 0,049) após a realização do *workshop* e treinamento com o seu ambiente de simulação de baixa fidedignidade. Apesar de existir melhora do tempo de confecção da anastomose, esse fator não foi estatisticamente significativo (p = 0,07) quando comparado a antes e depois do treinamento em simulação de anastomoses.

A Tabela 3 traz um resumo dos estudos incluídos.

**Tabela 3:** Extração de dados dos trabalhos incluídos na revisão de escopo.

Autor	Ano	País	Desenho do estudo	Participantes	Conceito	Contexto	Desfechos avaliados	Principais resultados	Outros	Nível de evidência
Wilasrusmee et al.	2007	EUA/TH	Prospectivo experimental, unicêntrico	38 residentes de CG (1 CV)	Avaliação da confecção de anastomose	Ambiente simulação	Tempo de sutura; vazamento; calibre da anastomose	Tempo de treinamento e tipo de anastomose são os únicos relacionados à diminuição do tempo de execução		1b
Duran et al.	2013	EUA	Prospectivo randomizado experimental, unicêntrico	92 residentes CV	Avaliação da confecção de anastomose	Ambiente simulação	*Checklist* da *performance* e OSATS para anastomose vascular	Pontuação do residente na escala global correlaciona-se com o seu nível de experiência		1b
Jogerst et al.	2021	EUA/TH	Prospectivo experimental, unicêntrico	12 residentes	Avaliação da confecção de anastomose	Vídeos gravados em ambiente de simulação	*Checklist* da *performance* global para OSATS	Variabilidade das classificações inter-examinador. Necessidade de treinamento para reprodutibilidade	Não foi descrita a especialidade do residente	1b
Torres et al.	2022	BRA	Prospectivo controlado, experimental unicêntrico	10 residentes de CV	Avaliação da confecção de anastomose	Ambiente simulação, após o *workshop*	OSATS; tempo e análise do produto final	Melhora dos resultados dos residentes após o *workshop*	Durante pandemia de covid-19	1b

EUA = Estados Unidos da América; TH = Tailândia; BRA = Brasil; CG = cirurgia geral; CV = cirurgia vascular; OSATS = *Objective Structured Assessment of Technical Skill.*

## DISCUSSÃO

A avaliação das habilidades cirúrgicas é de extrema importância na formação do cirurgião^1-6^ . Diferente do conhecimento cognitivo, que já possui múltiplos instrumentos validados de mensuração, as habilidades cirúrgicas estão bastante atrasadas quanto a suas possibilidades de quantificação^1^ . As anastomoses vasculares são procedimentos complexos e que precisam ser realizados perfeitamente, pois falhas podem comprometer a segurança do paciente, resultando em sangramentos ou tromboses^3^ . Esta revisão de escopo analisou os instrumentos de avaliação de anastomoses vasculares realizadas por residentes (treinandos) em cirurgia vascular no mundo. Até hoje, não há um instrumento validado e unificado para que serviços e residentes possam ser apreciados globalmente e, assim, esses dados possam ser comparados e estandardizados^5^ .

Todos os estudos incluídos nesta revisão avaliaram residentes apenas em ambiente de simulação. Não houve um estudo que tenha utilizado a sala de cirurgia real como o local de ensino e avaliação, apesar de ser essa a ideia inicial desta revisão. Dos quatro trabalhos incluídos nesta revisão, três utilizaram o OSATS para parte da avaliação das habilidades cirúrgicas^2,3,5^ . O OSATS é um instrumento já bastante estudado e validado mundialmente, inclusive no Brasil^7^ . Esses estudos incluíram *checklists* de desempenho adicionais para compreender os passos específicos de uma anastomose vascular, uma vez que o OSATS não contempla alguns desses passos^1^ .

O OSATS originalmente descrito avalia sete passos e encontra-se detalhado na Tabela 1. Cada um dos itens deve ser pontuado numa escala de 1 a 5, sendo 1 o pior desempenho e 5 o melhor^6^ . Diferente do OSATS já publicado, que apresenta modificações para laparoscopia, as modificações para a apreciação de uma anastomose não foram incluídas num OSATS específico para esse fim^13^ .

Os trabalhos utilizaram escalas paralelas para suprir a deficiência das avaliações dos passos específicos de uma anastomose. Torres et al.^2^ adicionaram a análise do produto final e o tempo de realização do procedimento ao OSATS tradicional. Jogerst et al. utilizaram o OSATS adaptado e resumido^5^ . Já Wilasrusmee et al.^1^ não utilizaram o OSATS, optando por três objetivos: tempo de execução do procedimento, grau de vazamento e tamanho da anastomose. Wilasrusmee et al.^1^ acharam que quanto mais treinado era o residente, mais rápido ele concluía toda a tarefa. Esta revisão julga muito importante esse tópico, mas sugere que ele seja analisado separadamente da lista de verificação. Isso porque há anastomoses mais difíceis de serem realizadas, que demandam mais tempo para a sua conclusão, como quando há a necessidade de fixação de placas ou descolamentos de íntima, sem que isso represente, por si só, uma deficiência no tempo de confecção.

Duran et al.^3^ foram os únicos a utilizar um instrumento de avaliação global para a avaliação do desempenho das anastomoses término-terminais. Eles acrescentaram hemostasia, sutura e nó e qualidade do produto final, e retiraram conhecimento do instrumental e conhecimento específico do procedimento. Eles próprios haviam sugerido uma lista de apreciação ainda maior, com 25 itens, mas concordaram no seu próprio trabalho que seria cansativo e de difícil reprodutibilidade^3^ .

Os autores desta revisão concordam que o OSATS é um brilhante instrumento para avaliação de habilidades cirúrgicas, sendo o melhor publicado até o momento. Entretanto, o instrumento precisa de adaptações para a realidade de cada procedimento cirúrgico. Após realizar esta revisão, sugerimos que ao OSATS original sejam acrescidos os seguintes tópicos para o julgamento de uma anastomose na sala de cirurgia: tamanho da anastomose, grau de vazamento e tempo para concluir o procedimento. Wilasrusmee et al.^1^ observaram que cada décimo de milímetro aumentado na arteriotomia estava drasticamente relacionado à queda de vazamentos. Como sugerido por Duran et al.^3^ , também julgamos vantajoso incluir hemostasia (de todos os tecidos, e não só da anastomose em si), sutura e nós e qualidade do produto final. Porém, diferente desses autores, nossa impressão é de que a sutura e o nó devam ser separados em tópicos diferentes, já que avaliam ações diferentes. Esta revisão de escopo sugere ainda que o tópico conhecimento específico sobre o procedimento seja retirado da lista de avaliação das habilidades cirúrgicas, já que a arguição sobre esse tópico é melhor realizada quando são aplicados testes cognitivos de conhecimentos teóricos. A pontuação de cada habilidade pode ser realizada pela escala de cinco pontos de Likert^13^ . O instrumento sugerido nesta revisão de escopo encontra-se apresentado na Tabela 4. A pontuação dos treinandos pode variar de 12 (pior desempenho) até 60 (melhor desempenho).

**Tabela 4:** Instrumento sugerido para avaliação de habilidades cirúrgicas em anastomoses vasculares.

	1	2	3	4	5
Respeito pelos tecidos	Frequentemente usa força desnecessária nos tecidos, causando dano pelo uso inapropriado de instrumentos		Manejo cuidadoso dos tecidos, mas ocasionalmente causa dano inadvertido aos tecidos		Consistentemente manuseia adequadamente os tecidos, com mínimo dano
Movimentação e tempo cirúrgico	Muitos movimentos desnecessários		Movimentos e tempo efetivos, mas com movimentos desnecessários		Economia de movimento, com o máximo de eficiência
Hemostasia	Pobre controle do sangramento por método inadequado ou causando dano tecidual		Alguns lapsos na hemostasia		Controle rápido do sangramento por método apropriado
Manuseio dos instrumentos	Faz tentativas repetidas ou movimentos inábeis com instrumentos		Uso competente de instrumentos, apesar de ocasionalmente aparentar grosseiro ou inábil		Movimentos fluidos com instrumentos hábeis
Conhecimento do instrumental	Frequentemente pede instrumento errado ou usa instrumento inapropriado		Sabe do nome da maioria dos instrumentos e usa o instrumento apropriado para a tarefa		Obviamente familiarizado com os instrumentos necessários e seus nomes
Utilização de assistentes	Posiciona mal os assistentes ou falha em seu uso		Bom uso dos assistentes na maioria do tempo		Utiliza o assistente estrategicamente bem em todo o tempo
Sutura	Má técnica causando mal aposição dos tecidos e distância dos pontos		Sutura confiável, mas realizada por movimentos inadequados		Tecidos bem posicionados, técnica suave e distância adequada
Nós	Número de nós insuficientes e frouxos		Número de nós suficientes, porém frouxos		Seis nós e tensão suficiente para manter a sutura
Vazamento	Anastomose não concluída	Líquido vazando por toda a anastomose	Líquido esguichando pela anastomose	Líquido pingando da anastomose	Sem vazamentos
Diâmetro da anastomose	1x o diâmetro do vaso		2x o diâmetro do vaso		1,5x o diâmetro do vaso
Fluxo da cirurgia e planejamento de passos	Frequentemente interrompe a cirurgia ou precisa de discutir o próximo passo		Demonstra habilidade de planejar o próximo passo com progressão estável da cirurgia		Obviamente planeja o curso da cirurgia sem esforço no passo a passo
Qualidade do produto final	Bem abaixo do esperado e com aparência de falha		Produto tem aparências de falhas, mas pode funcionar adequadamente		Excelente produto final, sem imperfeições e funcionando bem
Tempo total da anastomose					

Mínimo de 12 pontos: pior qualificação. Máximo de 60 pontos: residente atingiu a excelência na realização da tarefa.

Ao iniciar a pesquisa sobre o tema em questão (instrumento de avaliação de habilidades para residentes de cirurgia vascular), os autores imaginavam que poderiam existir poucas publicações sobre o assunto. Iniciada a pesquisa, foi descoberto que nenhuma síntese de evidência, estudo ou análise havia sido publicada ou estava em andamento no momento da coleta de dados. A revisão de escopo foi o método escolhido para a revisão da literatura porque ela tem a finalidade de procurar e analisar lacunas do conhecimento. As revisões de escopo partem de questionamentos mais amplos que as revisões sistemáticas e pretendem identificar gargalos que requeiram uma investigação futura. Além disso, são diferentes da revisão integrativa que, segundo Galvão e Pereira, reúne pesquisas de naturezas metodológicas distintas para uma compreensão mais abrangente de um fenômeno de saúde. Muito usado na área da enfermagem, esse tipo de revisão pode incluir pesquisas pré-clínicas e clínicas, qualitativas e quantitativas, em seus estudos elegíveis. Por meio da compatibilização de informações de naturezas diversas, seu propósito é apresentar a síntese de diferentes tipos de conhecimento sobre o assunto^14^ . A revisão integrativa possui um método pouco padronizado e corresponderia a um tipo de revisão narrativa. Por isso, procedeu-se uma revisão baseada no protocolo do JBI, utilizando o PRISMA Extension for Scoping Reviews (PRISMA-ScR) para reportar os resultados, com quadros apresentando o sumário dos estudos, acompanhado de uma síntese descritiva^14-16^ .

São várias as limitações deste estudo. Primeiro, a revisão de escopo foi realizada apenas numa base de dados, a PubMed, pois o tempo para realizá-la foi extremamente escasso. Foi escolhida a PubMed por se tratar da principal base de dados utilizada na medicina.

Segundo, todos os trabalhos incluídos foram realizados em ambiente de simulação. Não houve um estudo encontrado que avaliasse a confecção de anastomoses vasculares em tempo real, com o paciente, na sala de cirurgia. Isso porque estressores são adicionados ao procedimento real e não ao simulado, como: a hora do dia; pequenas diferenças nos instrumentos disponíveis; problemas clínicos do paciente; problemas em outras áreas dos hospitais; dificuldades inesperadas na cirurgia; *bip* do aparelho de celular, entre outros. Infelizmente, os laboratórios de simulação ainda são escassos no Brasil. É necessário validar instrumentos de aferição de habilidades cirúrgicas em ambientes reais de sala de cirurgia, correspondendo à realidade da maioria das residências de cirurgia vascular do Brasil, onde não há laboratórios de simulação disponíveis. A avaliação em ambiente real possibilitará a vantagem da apreciação de um item que não pode ser classificado no ambiente simulado, a hemostasia^5^ .

Por último, mesmo nos estudos incluídos, houve viés de seleção, pois um deles incluiu apenas um residente de cirurgia vascular^1^ , e outro, apesar de se tratar de um trabalho com residentes da área cirúrgica, não identificou com clareza a especialidade. No entanto, a utilização do OSATS em seu desenho e a avaliação das anastomoses vasculares por cirurgiões especialistas em cirurgia vascular fez com que o mantivéssemos entre os trabalhos incluídos^5^ .

As avaliações de habilidades cirúrgicas estão muito atrasadas quando comparadas às disponíveis para quantificar o conhecimento técnico-cognitivo. Poucos instrumentos para ponderação de habilidades estão disponíveis na literatura, sendo o OSATS o mais utilizado^6^ . Quando se fala em um instrumento específico para a confecção de uma anastomose vascular, o OSATS é frequentemente utilizado com pequenas alterações ou *checklists* distintos, mas sem validação ou utilização global.

Faz-se necessário desenvolver um instrumento para que seja possível uniformizar as análises dos residentes de cirurgia vascular e, consequentemente, seus programas de residência. Não há, no Brasil, qualquer instrumento desenvolvido com tal finalidade.

## CONCLUSÃO

O presente estudo identificou que há poucos trabalhos na literatura mundial utilizando instrumentos de avaliação de habilidades cirúrgicas relacionadas à realização de uma anastomose vascular.

No Brasil, a presente revisão encontrou um único estudo utilizando o OSATS e acrescentando o tempo para a confecção da anastomose e análise do produto final.

Todos os estudos utilizaram como cenário o ambiente de simulação, não havendo nenhum sequer que utilizasse instrumentos de avaliação de habilidades cirúrgicas no ambiente real da sala de cirurgia.

Esta revisão sugere um novo instrumento avaliatório de anastomoses vasculares a ser utilizado na sala de cirurgia.
